# Does Hepatosteatosis Affect Shear-Wave Elastography Values in Chronic Hepatitis Patients with the Same Histological Fibrosis Grade?

**DOI:** 10.3390/diagnostics16132053

**Published:** 2026-06-30

**Authors:** Emrah Karatay, Abdulkadir Eren

**Affiliations:** 1 Department of Radiology, Sultan II. Abdulhamid Han Training and Research Hospital, Istanbul 34668, Türkiye; emrahkaratay1984@gmail.com; 2 Department of Radiology, Istanbul Medipol University, Medipol Mega University Hospital, Istanbul 34214, Türkiye

**Keywords:** hepatosteatosis, chronic hepatitis, shear-wave elastography, Ishak, Knodell

## Abstract

**Background/Objectives**: The Ishak and Knodell classifications are used to evaluate fibrosis on percutaneous liver biopsy (PLB) in chronic hepatitis (CH). Hepatosteatosis (HS) can also be seen in PLBs performed on patients. In addition to PLB, the shear-wave elastography (SWE) technique is increasingly used to evaluate liver fibrosis. Whether the presence of HS contributed to fibrosis in CH cases that underwent PLB was evaluated by comparing SWE and Ishak–Knodell grades. **Methods**: CH cases were divided into three groups based on the presence of HS on biopsy and the percentage of HS (none, <10%, or ≥10%). Ishak–Knodell fibrosis grades and SWE values in kilopascal (kPa) were available for all included cases. Fibrosis scores and kPa values were compared within themselves and between groups for all three groups. Whether steatosis affected kPa results within the same fibrosis grades was evaluated. **Results**: There were 236 patients with CH: 109 (46.2%) had HS on liver biopsy, and 127 (53.8%) did not. Among the 109 patients with HS, 78 (71.5%) had pathological steatosis ≥ 10%. In all Ishak and Knodell grades (except F3), there was no significance between the liver stiffness values (kPa) of those with ≥10% HS and those with HS < 10%/none (*p* > 0.05). For the Ishak–Knodell F3 grade, there was a significant difference in both comparisons (*p* < 0.05). **Conclusions**: Liver kPa results are compatible with fibrosis grades, and SWE can be easily applied in the follow-up of CH patients with HS. In this way, unnecessary invasive procedures are prevented, and patient comfort can be maintained.

## 1. Introduction

Chronic hepatitis (CH) is a major global public health problem causing morbidity and mortality. It mostly develops due to viral causes. Hepatitis B virus (HBV) and hepatitis C virus (HCV) infections are the most common causes of CH in many countries. Approximately 400–500 million people are currently infected with HBV and HCV [1]. Viral hepatitis also causes cirrhosis, hepatocellular carcinoma, or liver failure, killing more than 1 million people each year. The regions where viral hepatitis is most common worldwide include Southeast Asia, Sub-Saharan Africa, Eastern Europe, and South America [2]. Autoimmune hepatitis (AIH) is another main cause and is suggested to be the cause of 10–20% of chronic hepatitis developing in Northern European, American, and Caucasian races [3].

The most important complications of CH are fibrosis and cirrhosis. Fibrosis is a result of chronic damage, and attacks and remissions during CH trigger its formation. Apart from being a direct indicator of liver damage, developing fibrosis also plays an important role in the development of hepatocyte dysfunction and portal hypertension [4]. In cases of CH, the level of disease parallels the degree of fibrosis. Knowing the status and progression of fibrosis is the most important data in evaluating the severity and prognosis of the disease and the response to treatment [5]. Therefore, patients with fibrosis benefit significantly from medical treatment when detected at an early stage. In advanced stages, drug treatment is insufficient in a significant number of cases, and even liver transplantation may be required [4,5]. In addition to fibrosis, hepatosteatosis (HS) may also accompany CH cases. HS has been shown to be more common in CH than in the general population [6]. This condition is defined as excessive lipid accumulation in the hepatocyte cytoplasm. Most researchers define this condition as triglyceride content exceeding 5% of the liver weight. The prevalence of HS varies between 35% and 81% in chronic hepatitis C [7].

Percutaneous liver biopsy (PLB) is currently used as the gold standard in the diagnosis of CH. Grading systems have been developed for the biopsy samples taken. The most used grading systems include Ishak, Knodell, METAVIR, and Scheuer scoring [8]. In the scoring suggested by the Knodell system, necrosis, inflammation, and fibrosis are examined. During this scoring, the fibrosis score is also specified, and staging is done. The Ishak scoring system is an update of the Knodell [9]. Although PLB is still the gold standard, the fact that the procedure is painful and complications can develop poses a significant problem. It is difficult to repeat in cases with long-term follow-up, and it poses a risk for the patient and the physician because it is an invasive method. There are also limiting factors such as sampling error and differences in evaluation between pathologists examining the biopsy material [10]. When it comes to imaging modalities, routine transabdominal ultrasonography (USG) is used in the initial evaluation of CH cases. USG has high sensitivity and specificity in the demonstration of liver disease, especially advanced disease and portal hypertension. However, it is not very successful in evaluating mild or moderate fibrosis [11].

In recent years, elastography applications have been used to evaluate CH and liver fibrosis. Although transient elastography (TE) is the most studied method for measuring liver elasticity, shear-wave elastography (SWE) is a newer technique. SWE can also be used to delineate focal hepatic lesions, estimate liver morphological changes, and monitor blood flow changes. It is also insensitive to the acid effect compared to TE [12]. Unlike the single-point measurement in PLB, in the SWE technique, multiple regions of interest (ROIs) are placed in the liver parenchyma, and quantitative measurements are made from multiple points. Thus, a wider area of the liver can be evaluated with simultaneous B-mode USG guidance [13]. The shear-wave velocity is measured from consecutive measurement points and then converted to Young’s modulus and reported in kilopascal (kPa). The overall accuracy of SWE in staging liver fibrosis is high, and it is an ideal method for the noninvasive assessment of liver fibrosis severity [12,13,14].

Whether the presence of HS contributes significantly to fibrosis scores in biopsied CH cases has not been sufficiently investigated to date [15,16,17,18,19,20]. In this study, whether the presence of HS affects Ishak–Knodell fibrosis stages and SWE measurements in CH cases was investigated. Thus, the effectiveness of SWE was evaluated by comparing it with fibrosis stages.

## 2. Materials and Methods

### 2.1. Study Design

This retrospective observational study protocol was approved by the clinical medical research ethics committee of the tertiary health center (IRB: E-10840098-202.3.02-1256). Informed consent forms were available for all participants who underwent biopsy and SWE. Patients who were referred to the radiology unit with the diagnosis of CH between May 2023 and January 2025 and who subsequently underwent PLB and SWE, respectively, within 6 months were evaluated retrospectively. Patients with chronic viral hepatitis and AIH in the age group ≥ 18 years were included. Patients with body mass index > 40, younger than 18 years, history of hepatobiliary surgery, transplanted liver, concomitant mass lesions in the liver, undergoing antiviral therapy, and those unable to hold their breath were excluded. Patients’ characteristics, SWE data, and PLB results were recorded. The cases were divided into three groups according to the presence and percentage of HS: group A (none), group B (HS < %10), and group C (HS ≥ %10). This limit was used because at least 10% of the parenchyma must be affected to mention diffuse HS [15,20]. Using group C as a limit, groups B-C and then A-C were compared among themselves. In this way, the effects of HS absence, focal HS, and diffuse HS on SWE were evaluated.

### 2.2. SWE Imaging and Measurements

**Figure 1:**
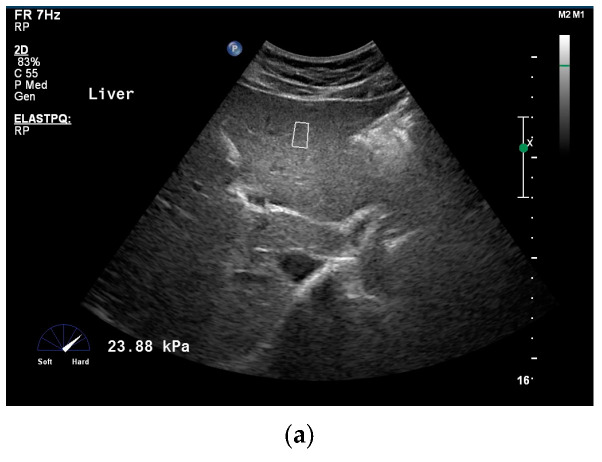

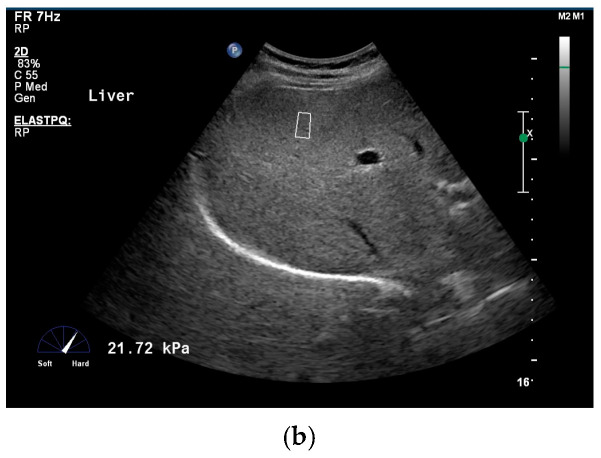
In a 36-year-old male patient with histopathologically proven absence of hepatosteatosis, measurements were taken in kPa using standard ROI with the SWE technique at liver segments 2 (**a**) and 8 (**b**) (ROI is represented by quadrangular shapes with white edges).

**Figure 2:**
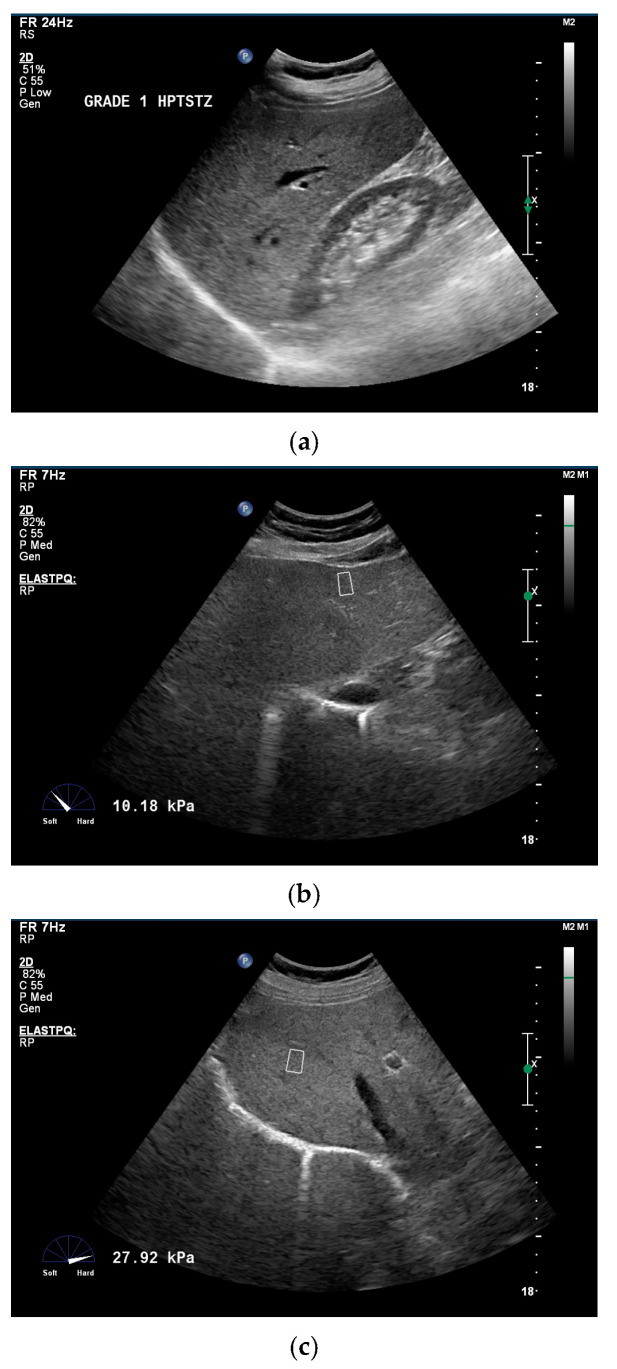
In a 44-year-old female patient with histopathologically proven hepatosteatosis (HS < 10%), grade I hepatosteatosis and enlargement of the left lobe lateral segment were observed in B-mode US (**a**). Measurements in kPa were taken in segments 2 (**b**) and 7 (**c**) of the liver using the SWE with standard ROI (ROI is represented by quadrangular shapes with white edges).

All measurements were performed by a practitioner experienced in abdominal radiology. A 1–5 MHz convex USG probe (Philips IU22, Bothell, WA 98041, USA) was used during the procedures. Since hepatobiliary USG was performed before SWE, and B-mode imaging was used as a guide during the measurements, the patient was asked to fast for at least 8 h before the procedure. USG and SWE procedures were performed in the supine position and left decubitus position with the patient’s right arm in full abduction by holding the patient’s breath from the intercostal and subcostal spaces at the level of the subxiphoidal line, midclavicular line, and midaxillary line. After switching to SWE mode by pressing the button on the device, shear waves were composed by applying pressure to the skin with the ultrasound probe. In each case, measurements were made from a total of 8 different points in the liver using the Couinaud classification, two of which were from the left lobe and two from the caudate lobe (Figure 1). The measurement values obtained by placing a standard ROI (15 × 10 mm) on each segment of the liver were considered. When placing the ROI measurement points, vascular structures were avoided as much as possible. After each point measurement, values in kPa were obtained (Figure 2). All values were obtained from the liver parenchyma up to 8 cm in depth.

### 2.3. Histopathological Staging

Fibrosis scores of biopsy materials were determined using the Ishak and Knodell classifications [8,9]. According to Knodell, biopsy samples were graded into four categories (F0-1-3-4). They are listed as F0: no fibrosis; F1: fibrous portal expansion; F3: bridging fibrosis (portal–portal or portal–central linkage); and F4: cirrhosis. Ishak scoring is a modified version of the Knodell scoring system and had 6 categories (F0-1-2-3-4-5-6). It is graded as F0: no fibrosis; F1: fibrous expansion of some portal areas, with or without short fibrous septa; F2: fibrous expansion of most portal areas, with or without short fibrous septa; F3: portal fibrous with occasional (portal–portal bridging); F4: portal fibrosis with marked (portal–portal bridging or portal–central bridging); F5: marked bridging with occasional nodules (incomplete cirrhosis); and F6: probable or definite cirrhosis. F5 and F6 reflect incomplete and definite cirrhosis, respectively. This system also helps to determine the levels of necro-inflammatory activity through grading.

### 2.4. Statistical Analysis

Data analysis was performed using IBM SPSS ver. 25 (Armonk, NY, USA) program. The Kolmogorov–Smirnov test was used to analyze the distribution of data. Since elasticity values (kPa) did not show a normal distribution, non-parametric tests were used, and quantitative SWE values were reported as the median. Descriptive statistical methods (median, frequency, percentage, interquartile range (IQR), minimum, and maximum) were used to evaluate and express central tendency. Continuous variables were compared using the Mann–Whitney U test, and categorical variables were compared using the chi-squared test or “Fisher’s exact test”. The correlation between elasticity values and histological fibrosis stages was evaluated with the help of the Spearman correlation test. A 95% confidence interval was accepted in all tests, and *p* < 0.05 was considered statistically significant.

## 3. Results

A total of 236 patients were included, 91 were female and 145 were male. The youngest patient was 18 years old, and the oldest patient was 73 years old, both male. For females, the youngest case was 21 and the oldest case was 71 years old. The mean age for all cases was calculated as 38.12 ± 7.46. A total of 169 patients (71.6%) had chronic hepatitis B (CHB), 44 patients (18.7%) had chronic hepatitis C (CHC), and 23 patients (9.7%) had AIH. When the relationship between the causes of CH and gender was evaluated, no significant difference was found (*p* > 0.05). There was also no significant relationship between age and gender (*p* > 0.05).

All cases were evaluated according to the Ishak and Knodell histopathological fibrosis grading systems. According to the Knodell classification, 40 patients (16.9%) were defined as F0, 103 (43.6%) as F1, 72 (30.6%) as F3, and 21 (8.9%) patients as F4. In the Ishak classification, 41 patients (17.4%) were defined as F0, 58 (24.6%) as F1, 46 (19.4%) as F2, 51 (21.6%) as F3, 21 (8.9%) as F4, 12 (5.1%) as F5, and seven patients (3.0%) as F6 (Table 1). According to the Ishak classification, significant fibrosis is defined as Ishak F ≥ 2, and 137 (58.1%) of the patients were compliant with this criterion. There was no significant relationship between F values and gender in both grading systems (*p* > 0.05).

In the evaluation for HS, 109 patients (46.2%) had steatosis in the liver biopsy pathology, while 127 (53.8%) did not have steatosis. According to the same pathology results, 78 patients (71.5%) had steatosis ≥ 10%. In other words, 78 of 236 patients (33.1%) had ≥ 10% steatosis in their pathology, and 158 (66.9%) had less than 10% steatosis. The relationship between the presence of HS and SWE results was evaluated. Therefore, whether there was a difference in SWE values between those with ≥10% HS and <10% (including those without steatosis) within the same histopathological grade was investigated. There was no significant difference between the SWE (liver stiffness) values of those with ≥10% steatosis and those with <10% in all Knodell and Ishak grades except F3. However, when the Mann–Whitney U test was used, there was a statistical difference within the Knodell–Ishak F3 grade (Table 2). Additionally, whether there was a difference in SWE values between ≥10% HS in the histopathological score and those without HS was also evaluated. In all Knodell and Ishak stages (except F3), there was no significant difference between the SWE (liver stiffness) values of those with HS ≥ 10% and those who did not have HS. When the Mann–Whitney U test is used only for the Knodell–Ishak F3 grade, there was statistical significance (Table 3). According to liver lobes (right and left), there was no significant difference between fibrosis grades and SWE values.

Spearman’s correlation was also used. Accordingly, there was a significant moderate (+) correlation between SWE (liver stiffness) values and Knodell grades (*ρ* = 0.405, *p* < 0.0001). So, as the fibrosis grade increases, liver stiffness also increases. Similarly, there was a significant moderate (+) correlation between SWE values (liver stiffness) and Ishak grades (*ρ* = 0.406, *p* < 0.0001).

## 4. Discussion

The observation that fibrosis can regress or improve with effective treatment in CH has led to new developments. Regardless of the cause and mechanism, liver fibrosis should be considered a dynamic process and should be monitored with diagnostic methods [21]. PLB is still considered the gold standard for the diagnosis and evaluation of liver fibrosis. However, it is a method that requires intervention, carries a risk of complications, requires a trained practitioner, and is costly [5,8,9]. There are also limiting factors such as sampling errors, noncompliant patients, and differences in evaluation among pathologists who examine biopsy material. Since it has been learned that CH is a dynamic process, it has become necessary to perform repeated PLBs on patients. The mentioned disadvantages have led researchers to use noninvasive methods for detecting liver fibrosis [10].

SWE is a noninvasive method in which images are obtained by evaluating the tissue response to localized mechanical stimuli sent via the USG probe. Its main advantages are that it allows imaging from multiple planes, does not use contrast agents, and does not contain ionizing radiation. SWE images and kPa values obtained under B-mode guidance provided a new opportunity to evaluate liver fibrosis [12,13]. The values of the speeds of the sliding waves created by mechanical stimuli in the tissue allow us to make quantitative estimates of tissue stiffness with the SWE technique [14]. Unlike the measurement made from a single point in PLB, there is also the advantage of placing as many ROI points as possible in the liver parenchyma, allowing measurements to be made from many points in the right and left liver lobes, thus evaluating a wider area [12,13,14].

There are a few studies evaluating the effect of HS on fibrosis using SWE in CH cases. Xie X et al. investigated whether HS affects the diagnostic accuracy of SWE in patients with CHB. Hepatic fibrosis and inflammatory activity degrees in 161 patients with CHB were staged according to the METAVIR classification. The extent of HS was defined as the percentage of hepatocytes containing fat droplets using oil red staining. SWE values were found to be independently associated with HS in the early stages of liver fibrosis (F0–F2 or F0–3). Furthermore, when HS ≥ 10%, SWE values were higher than the corresponding values in F0–2 (6.82 ± 1.57 vs. 7.92 ± 1.99; *p* = 0.010) and F0–3 (7.18 ± 1.84 vs. 8.25 ± 1.91; *p* = 0.007). It has been noted that the use of SWE in CHB patients with HS ≥ 10% overestimates the stage of fibrosis [15]. Ye J et al. evaluated the efficacy of SWE in 440 CHB (286 liver biopsy and 154 clinical decompensated cirrhosis) patients. HS was graded using a magnetic resonance imaging proton density fat fraction. It has been stated that HS is an independent factor in CHB patients, and its use, together with the SWE values, may be valuable in deciding on the PLB for fibrosis staging [16].

Shen F et al. hypothesized that moderate-severe HS may lead to the overestimation of SWE measurement in CHB patients without significant fibrosis. In CHB patients, HS was classified as absent (S0, <5%), mild (S1, 5–33%), and moderate-severe (S2–3, >33%), according to the pathology. Liver fibrosis was evaluated using METAVIR classification and SWE. There were 593 CHB cases in total, and the prevalence of HS was 37.6%. Without significant fibrosis (F0–1), the median SWE (kPa) was 7.4 at S2–3, which was significantly higher than the value at S0/S1 (*p* = 0.005). No significant difference was found in terms of significant fibrosis (F2–4). SWE has been shown to be useful for diagnosis in the presence of moderate-severe HS in F0–1 CHB patients [17]. Liu J et al. evaluated whether SWE values were affected by HS in CHB cases. HS and liver fibrosis were assessed by METAVIR grades and SWE. In patients with a low amount of fibrosis (F0–1 and F0–2), the median SWE was 8.8 kPa, and in patients with moderate-severe steatosis, it was 9.2 kPa (*p* < 0.05). It was also stated that to avoid overestimation of SWE, the presence of moderate-severe HS detected by histopathology should be considered [18].

Huang Z et al. evaluated the diagnostic performance of SWE in CHB patients affected by different HS stages and fibrosis. A total of 1306 patients were included and METAVIR scoring was used for fibrosis. The HS classification was divided into none (S0, <5%), mild (S1, 5–33%), and moderate-severe (S2–3, >33%). There was no significant difference in the median SWE values in F0–1 patients with different expected HS grades (*p* > 0.05). Moderate-severe HS in ≥ F2 affected SWE values in CHB patients, resulting in a less accurate diagnostic performance [19]. Conti F et al. studied the accuracy of SWE for the diagnosis of liver fibrosis in 211 cases with CHC and the effect of HS on liver stiffness measurement. Ishak classification was used for fibrosis grading, and HS was mentioned as absent (<5%), present (≥5%), or significant (≥10%), according to the percentage of fat content in hepatocytes. Among patients with the same fibrosis stages (F0–2 and F3–6; F0–3 and F4–F6), mean SWE values were similar in patients with HS (≥10% detected by PLB or USG) compared to absent HS. The discordance between the SWE measurement and histopathology was affected by a BMI > 30 kg/m^2^ (*p* < 0.05), and the presence of obesity was found to lead to the misclassification of significant-advanced fibrosis [20].

Unlike CHB or CHC in previous studies, CHB, CHC, and AIH cases were evaluated together in our study [15,16,17,18,19,20]. Thus, the effectiveness of HS in fibrosis was evaluated for different CH causes. While almost all other studies used only the METAVIR system for fibrosis definition, the Ishak and Knodell systems were used in our study [15,16,17,18,19]. Thus, SWE values were compared with more fibrosis stages and whether HS was effective was investigated in more detail. As a result of the use of different fibrosis scoring systems, it was attempted to determine which scoring system would be more appropriate in the evaluation of SWE results. In a previous study, although only Ishak classification was used, the HS staging was different from our study and included only CHC cases [20]. Different from the literature, our results suggested that the effectiveness of HS was significant only for the Ishak–Knodell grade F3. HS may additively increase apparent stiffness specifically at the bridging fibrosis stage, where periportal fibrous septa and lipid-laden hepatocytes coexist without the dominant mechanical effect of the mature cirrhotic matrix. This provides a sufficient basis to construct a partly speculative, but consistent, mechanistic argument to understand why there is no significant difference between Ishak and Knodell in F3 grade cases [15,17,18,19].

This study had several limitations. The first limitation of our study was that SWE procedures were performed by one radiologist, and therefore, interobserver comparisons could not be made. The second limitation was that there were few studies evaluating the effectiveness of HS in CH cases with which we could compare our SWE results [15,16,17,18,19,20]. The third limitation was the lack of a healthy control group. The fourth limitation was that the dimensions of the ROI point measured during the examination could not be changed. The fifth limitation is that multiple independent statistical tests were performed across Knodell and Ishak fibrosis grades without any comparison correction for multiplicity. This leads to a relatively incomplete interpretation of the data. Another limitation is the lack or absence of some data that can be used for comparison during archival research (BMI within the non-excluded range, ALT at the time of SWE, necro-inflammatory grade, and etiological subgroup).

## 5. Conclusions

In contrast to the measurement made from a single point in PLB, the SWE method has the advantage of placing as many ROI points as possible in the liver parenchyma. This allows measurements to be made from many points in the liver lobes and evaluates a wider area of the liver. In a situation where it is known that CH is a dynamic process and most etiologies of hepatitis are known to be lifelong, applying the SWE method to patients instead of repeated PLB is a much simpler and wiser choice. In our study, the fact that liver stiffness values were only affected for F3 showed that SWE can be used effectively in the follow-up of CH patients with HS. However, new studies including multiple fibrosis scoring systems and different causes of CH are needed to verify our results.

## Figures and Tables

**Table 1:** Fibrosis classifications and patient distribution.

	Knodell Scoring System				Ishak Scoring System		
Total Patients	Fibrosis (F) Grade	*n*	Percentage (%)	Total Patients	Fibrosis (F) Grade	*n*	Percentage (%)
*n*: 236	F0	40	16.9	*n*: 236	F0	41	17.4
					F1	58	24.6
	F1	103	43.6		F2	46	19.4
					F3	51	21.6
	F3	72	30.6		F4	21	8.9
					F5	12	5.1
	F4	21	8.9		F6	7	3.0

**Table 2:** Fibrosis grades and SWE values according to HS percentage.

**Knodell System Grades**	**Percentage (%) Number**	**SWE Value (kPa); Median and (IQR)**	***p*** -Value (* <0.05, ** <0.001)
F0 (*n*: 40)	HS < 10% (*n*: 26)	10.2 (5.1)	0.308
	HS ≥ 10% (*n*: 14)	13.3 (12.6)	
F1 (*n*: 103)	HS < 10% (*n*: 77)	11.9 (5.2)	0.429
	HS ≥ 10% (*n*: 26)	11.7 (6.8)	
F3 (*n*: 72)	HS < 10% (*n*: 47)	12.5 (4.9)	0.012 *
	HS ≥ 10% (*n*: 25)	17.4 (9.1)	
F4 (*n*: 21)	HS < 10% (*n*: 9)	30.7 (12.8)	0.692
	HS ≥ 10% (*n*: 12)	28.6 (26.9)	
**Ishak System Grades**	**Percentage (%) Number**	**SWE Value (kPa); Median and (IQR)**	***p*** -Value (* <0.05, ** <0.001)
F0 (*n*: 41)	HS < 10% (*n*: 26)	10.1 (5.3)	0.301
	HS ≥ 10% (*n*: 15)	13.2 (13.6)	
F1 (*n*: 58)	HS < 10% (*n*: 46)	11.9 (5.2)	0.427
	HS ≥ 10% (*n*: 12)	12.2 (10.1)	
F2 (*n*: 46)	HS < 10% (*n*: 31)	12.3 (4.2)	0.695
	HS ≥ 10% (*n*: 15)	12.9 (4.6)	
F3 (*n*: 51)	HS < 10% (*n*: 37)	11.3 (5.2)	0.003 *
	HS ≥ 10% (*n*: 14)	17.9 (11.7)	
F4 (*n*: 21)	HS < 10% (*n*: 11)	15.5 (6.3)	0.987
	HS ≥ 10% (*n*: 10)	15.7 (13.6)	
F5 (*n*: 12)	HS < 10% (*n*: 6)	27.6 (11.8)	0.686
	HS ≥ 10% (*n*: 6)	28.3 (21.4)	
F6 (*n*: 7)	HS < 10% (*n*: 4)	34.6 (13.9)	0.672
	HS ≥ 10% (*n*: 3)	31.2 (28.6)	

SWE, shear-wave elastography; HS, hepatosteatosis; F, fibrosis; and kPa, kilopascal.

**Table 3:** Fibrosis grades and SWE values according to HS presence and percentage.

**Knodell System Grades**	**Percentage (%) Number**	**SWE Value (kPa); Median and (IQR)**	***p*** -Value (* <0.05, ** <0.001)
F0 (*n*: 39)	None-HS (*n*: 25)	10.0 (6.3)	0.305
	HS ≥ 10% (*n*: 14)	13.2 (13.8)	
F1 (*n*: 86)	None-HS (*n*: 60)	11.8 (3.6)	0.268
	HS ≥ 10% (*n*: 26)	11.5 (6.6)	
F3 (*n*: 49)	None-HS (*n*: 24)	11.1 (5.2)	0.003 *
	HS ≥ 10% (*n*: 25)	17.3 (9.4)	
F4 (*n*: 31)	None-HS (*n*: 18)	33.9 (7.2)	0.448
	HS ≥ 10% (*n*: 13)	28.8 (26.5)	
**Ishak System Grades**	**Percentage (%) Number**	**SWE Value (kPa); Median and (IQR)**	***p*** -Value (* <0.05, ** <0.001)
F0 (*n*: 39)	None-HS (*n*: 23)	10.2 (6.1)	0.305
	HS ≥ 10% (*n*: 16)	13.2 (13.4)	
F1 (*n*: 51)	None-HS (*n*: 38)	11.8 (5.2)	0.362
	HS ≥ 10% (*n*: 13)	12.1 (9.8)	
F2 (*n*: 36)	None-HS (*n*: 21)	11.3 (3.6)	0.398
	HS ≥ 10% (*n*: 15)	11.4 (4.6)	
F3 (*n*: 42)	None-HS (*n*: 27)	10.5 (4.5)	0.001 **
	HS ≥ 10% (*n*: 15)	17.9 (11.8)	
F4 (*n*: 18)	None-HS (*n*: 8)	14.8 (7.2)	0.833
	HS ≥ 10% (*n*: 10)	14.6 (13.9)	
F5 (*n*: 12)	None-HS (*n*: 6)	26.7 (11.2)	0.418
	HS ≥ 10% (*n*: 6)	28.1 (21.1)	
F6 (*n*: 7)	None-HS (*n*: 4)	34.8 (7.8)	0.462
	HS ≥ 10% (*n*: 3)	29.1 (26.3)	

SWE, shear-wave elastography; HS, hepatosteatosis; F, fibrosis; and kPa, kilopascal.

## Data Availability

The data presented in this study are available on request from the corresponding author due to privacy restrictions.

## Abbreviations

CH	Chronic hepatitis
HBV	Hepatitis B virus
HCV	Hepatitis C virus
HS	Hepatosteatosis
PLB	Percutaneous liver biopsy
USG	Ultrasonography
TE	Transient elastography
SWE	Shear-wave elastography
ROI	Region of interest
kPa	Kilopascal
CHB	Chronic hepatitis B
CHC	Chronic hepatitis C
